# Exploring perfusion limits in pedicled perforator propeller flaps via intraoperative indocyanine green angiography

**DOI:** 10.1016/j.jpra.2026.07.016

**Published:** 2026-07-22

**Authors:** Adrian Sebald, Ingo Ludolph, Corinna Mundschau, Andreas Arkudas, Raymund E. Horch

**Affiliations:** Department of Plastic and Handsurgery and Laboratory for Tissue Engineering and Regenerative Medicine, University Hospital Erlangen, Friedrich-Alexander-Universität Erlangen-Nuernberg FAU, Chair. Univ.-Prof. Dr. Prof.h.c. Dr.h.c. Raymund E Horch, Krankenhausstrasse 12, 91054 Erlangen, Germany

**Keywords:** Indocyanine green angiography (ICG-A), Pedicled perforator propeller flap (PPF), Intraoperative perfusion assessment, Flap design optimization

## Abstract

**Background:**

Pedicled perforator propeller flaps are widely used for soft-tissue reconstruction because they preserve donor-site function while providing reliable defect coverage. However, intraoperative assessment of flap perfusion remains challenging, particularly in longer flaps or after axial rotation. Indocyanine green (ICG) fluorescence angiography allows real-time visualization of tissue perfusion and may improve intraoperative decision-making.

**Methods:**

A retrospective single-center study was performed including 30 patients who underwent reconstruction with pedicled perforator propeller flaps between 2014 and 2022. Intraoperative ICG angiography was performed using the SPY-Elite system after flap elevation and after inset. A total of 48 angiographic sequences were analyzed using ImageJ software. Fluorescence intensity, perfusion distribution, and contour levels (20% and 30%) were evaluated across standardized 3-cm flap segments from the perforator origin to the distal flap edge.

**Results:**

All flaps survived, resulting in a flap survival rate of 100%. Postoperative complications occurred in 6 patients (20%), including 3 cases of minor wound-healing disorders and 3 cases of venous congestion requiring revision. Mean flap length was 15.8 cm. The average perfused area at the 20% fluorescence contour level represented 97.2% of the total flap surface. Perfusion decreased progressively with increasing distance from the perforator and declined significantly in flaps exceeding 15 cm in length (*p* < 0.01). Flaps shorter than 10 cm demonstrated homogeneous perfusion across all segments. Lower extremity flaps showed more consistent perfusion patterns compared with truncal and gluteal flaps.

**Conclusions:**

Intraoperative ICG fluorescence angiography provides a reliable method for assessing perfusion in pedicled perforator propeller flaps. Perfusion mapping suggests that flap lengths under 10 cm and tissue areas within the 20%–30% fluorescence contour levels are associated with consistent viability. ICG angiography may therefore serve as a useful adjunct to optimize flap design and enhance surgical safety in reconstructive procedures.

## Introduction

Pedicled perforator propeller flaps (PPFs) have become an essential component of modern reconstructive surgery. By preserving the source vessel and minimizing donor-site morbidity, these flaps allow reliable coverage of soft-tissue defects while maintaining functional and aesthetic outcomes.^1^, ^2^, ^3^, ^4^ Since the introduction of the angiosome and perforasome concepts by Taylor,^5^ and Saint-Cyr,^6^ Hallock,^7^ and Rozen,^8^ a detailed understanding of perforator vascular territories has significantly advanced flap design and expanded the indications for local perforator-based reconstruction.

**Fig. 1:**
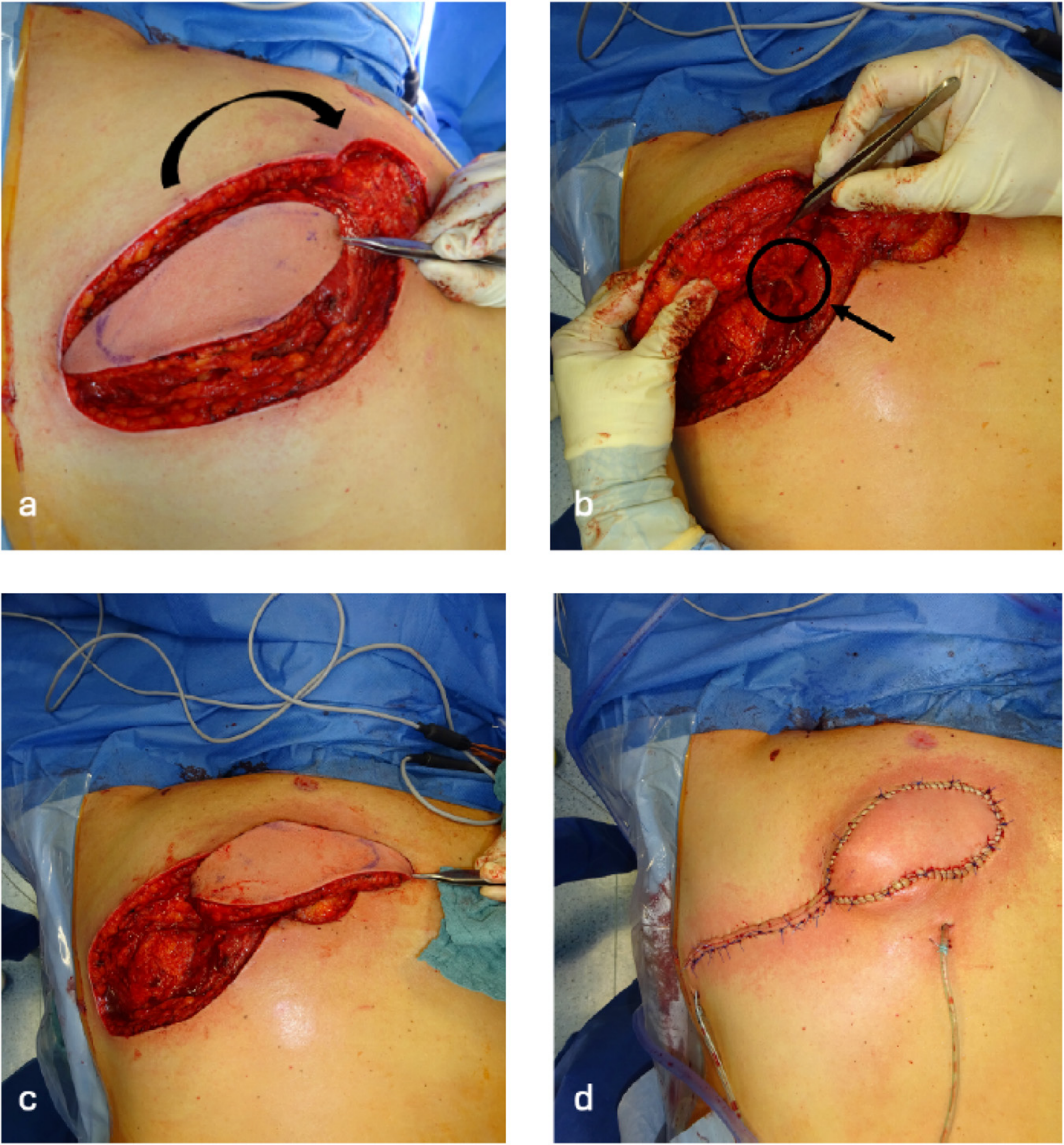
Intraoperative sequence of a pedicled perforator flap (a flap design with rotation planned around the perforator, b intraoperative view showing the dissected perforator vessel serving as the pivot point, c rotation and final transposition, d final inset with tension free closure).

In PPF surgery, a suitable perforator vessel is identified and meticulously dissected to its source while maintaining its vascular integrity. The flap is then designed around this perforator and rotated—often up to 180 degrees—to reach the defect (Fig. 1). The development of PPFs allows for efficient, tension-free closure of complex defects with axial rotation and precise contouring.^9^ albeit is not free of wound healing issues.^10^ Traditionally, intraoperative assessment of flap viability relies on clinical judgment, including evaluation of tissue color, capillary refill, dermal bleeding, and Doppler signals.^11^^,^^12^ While these methods remain widely used, they provide only indirect and often subjective information about tissue perfusion. Particularly in extended perforator flaps, defining the safe perfusion limits intraoperatively can be challenging.

We discuss the role of intraoperative Indocyanine green (ICG) fluorescence angiography which offers real-time, semi-quantitative perfusion assessment, addressing one of the key challenges in PPF surgery, if this is ensuring sufficient distal perfusion and reducing ischemia-related complications.^13^ Indocyanine green (ICG) fluorescence angiography has emerged as a valuable adjunct for intraoperative perfusion assessment. ICG is a near-infrared fluorescent dye that rapidly binds to plasma proteins and remains confined to the intravascular compartment until hepatic clearance. It has widespread uses in cardiology, ophthalmology and hepatology, and identifying sentinel lymph nodes in cancer staging. Its application in reconstructive surgery, especially for flap monitoring, is expanding.^14^ While subjective clinical evaluation and Doppler use remain standard, ICG imaging provides reproducible and reliable quantifiable data that may guide intraoperative decision-making, including flap design.

When visualized with near-infrared imaging systems, ICG allows dynamic, real-time visualization of tissue perfusion. In reconstructive surgery, this technique has been increasingly used to evaluate flap viability, guide flap design, and reduce ischemic complications.

### Aim of the study

The aim of this study was therefore to analyze intraoperative ICG-based perfusion patterns in pedicled perforator propeller flaps. Specifically, we sought to (1) evaluate perfusion distribution along the flap axis, (2) assess the influence of flap length and anatomical location on perfusion patterns, and (3) analyzse whether a 20%–30% fluorescence contour levels represents a reliable indicator of viable tissue in PPF reconstruction.

### Patients, methods and study design

A retrospective, single-center study was conducted at the Department of Plastic and Hand Surgery, University Hospital Erlangen, between November 2014 and June 2022.

Patients: The study cohort consisted of all consecutive patients who underwent reconstruction with a pedicled perforator propeller flap and in whom intraoperative ICG fluorescence angiography had been performed according to the institutional surgical protocol. Thirty patients (12 men, 18 women; age range: 24–87 years, supplementary 1) were included. Inclusion required intraoperative ICG angiography using the SPY-Elite system (Stryker, Michigan USA). Flap reconstruction was performed for a broad spectrum of soft-tissue defects, including oncologic defects following tumor resection, pressure sores, chronic wounds and fistulas associated with inflammatory bowel disease, postinfectious defects, and traumatic or postsurgical defects of the trunk and extremities. Postoperative follow-up was performed according to the institution’s standard clinical protocol, with regulary routine assessments until approximatly 12 months after surgery. During follow-up, flap viability, wound healing, and postoperative complications, including venous congestion, partial flap necrosis, and the need for revision surgery, were documented.

ICG Angiography Protocol: ICG (5 ml, 2.5 mg/ml) was injected intravenously under standardized OR conditions. Two measurements were taken: after flap elevation (H) and after flap inset (E). Maximum fluorescence frames were selected manually for analysis.

**Fig. 2:**
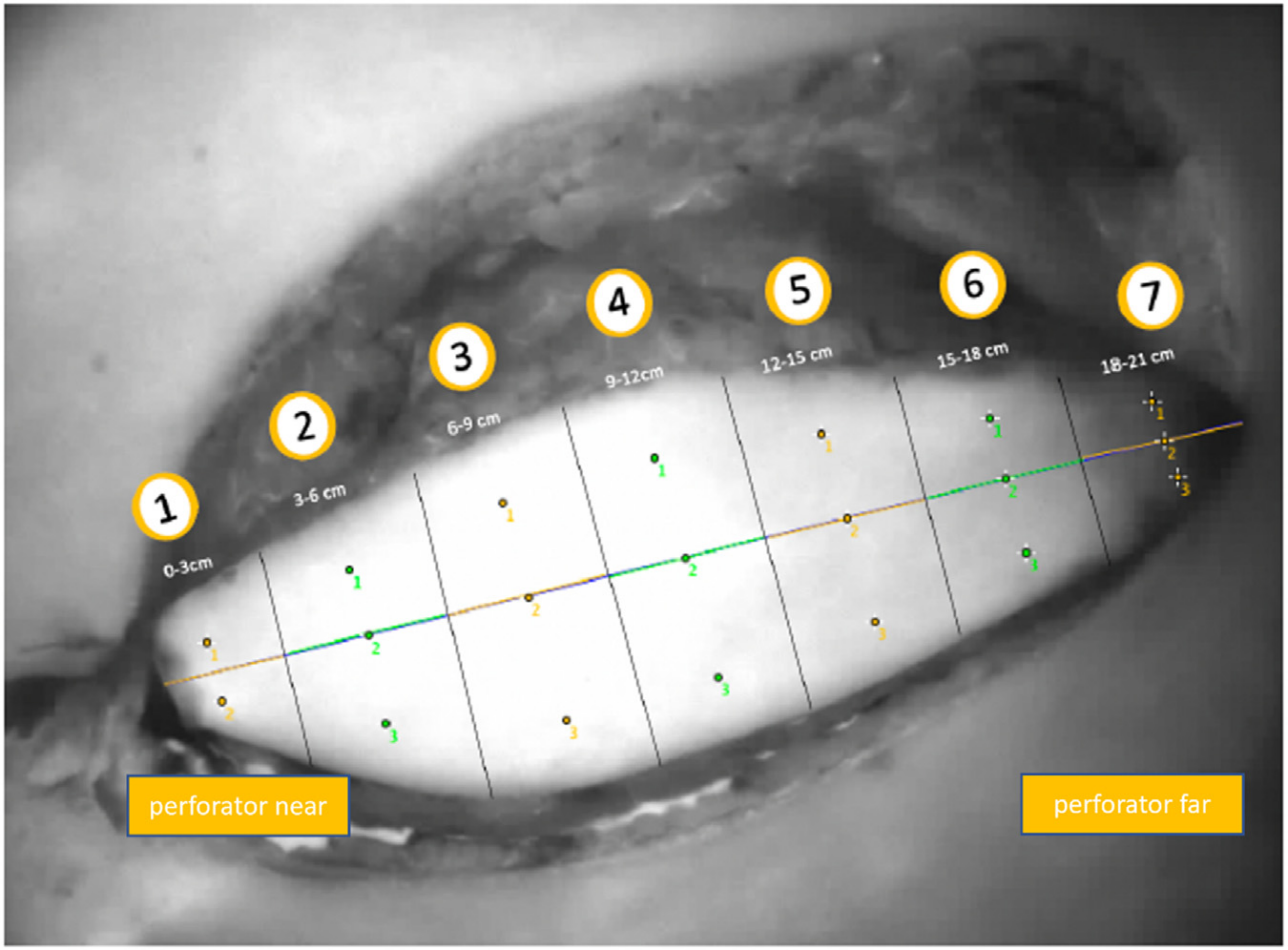
Perfusion drop-off >20 cm shown in ICG fluorescence map.

Image Analysis: ImageJ software was used to analyse fluorescence distribution. Each flap image was divided into 3-cm zones from the perforator origin to the most distant flap border. Fluorescence intensity and contour levels (20% and 30%) were measured. Contour levels define perfusions borders in relation to the point of maximum perfusion. A pixel-to-cm ratio was calibrated using intraoperative scale images (Fig. 2).

Statistical Analysis: Normal distribution was assessed using Shapiro-Wilk and Kolmogorov-Smirnov tests. ANOVA with Dunnett’s post-hoc test, paired *t*-tests, and Wilcoxon tests were used for group comparisons. Significance was set at *p* < 0.05.

## Results and overall outcomes

All 30 flaps survived, yielding a flap survival rate of 100%. A total of 6 patients (20%) experienced postoperative complications. Three complications were classified as minor and included wound healing disorders that resolved with conservative treatment. The remaining three complications were related to venous congestion requiring surgical revision, yet all flaps remained viable without partial necrosis (supplementary 1).

### Perfusion measurements

Forty-eight ICG sequences were evaluated. Mean flap length was 15.8 cm (range: 4.6–27.2 cm). The average perfused area at the 20% contour level was 91.5 cm², equating to 97.2% of the total flap area, indicating a high rate of viable perfusion even in extended flaps.

**Fig. 3:**
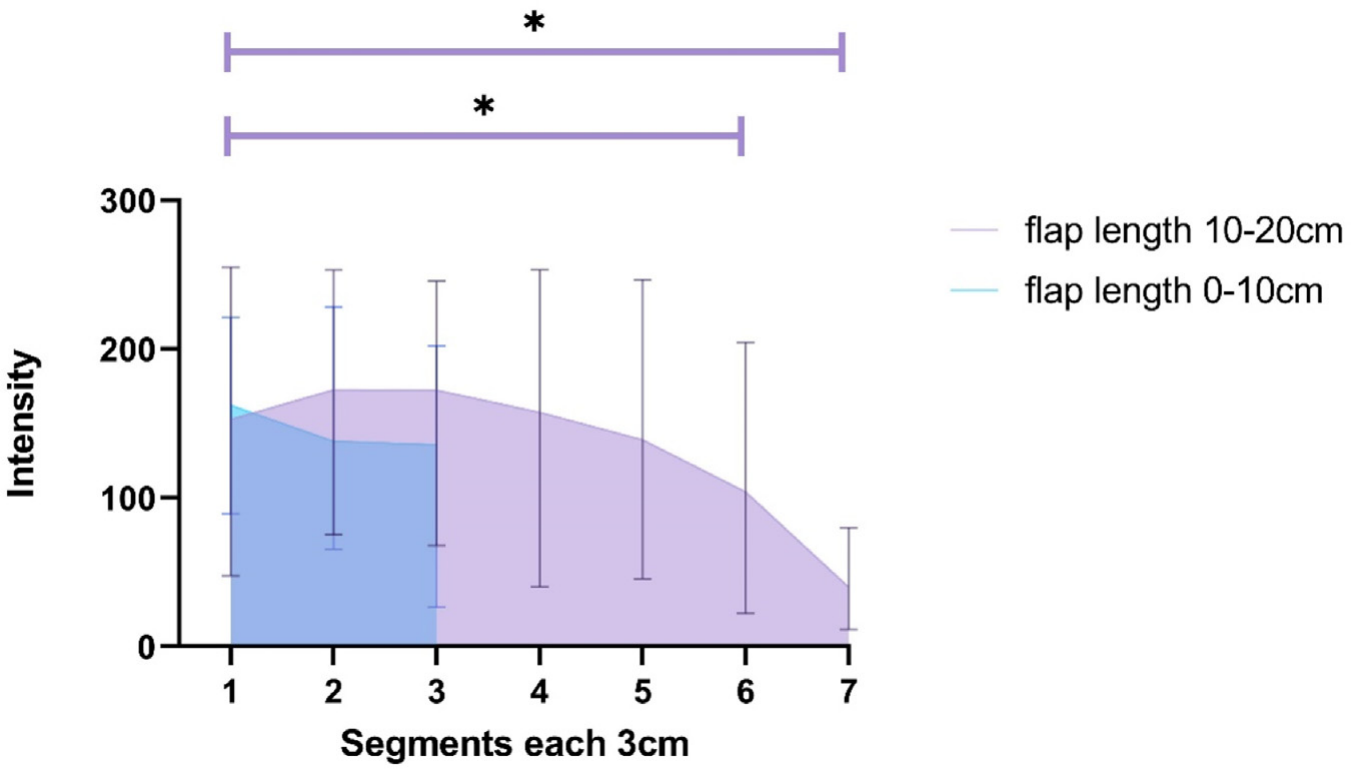
Perfusion analysis depending on flap length.

Perfusion analysis revealed a decreasing fluorescence intensity pattern with increasing distance from the perforator. Specifically, flaps greater than 15 cm in length showed significant declines in distal perfusion (*p* < 0.01). However, flaps measuring <10 cm demonstrated homogeneous perfusion across all measured segments with minimal fluorescence decrease (mean Δ intensity = 2.3 units; Fig. 3).

**Fig. 4:**
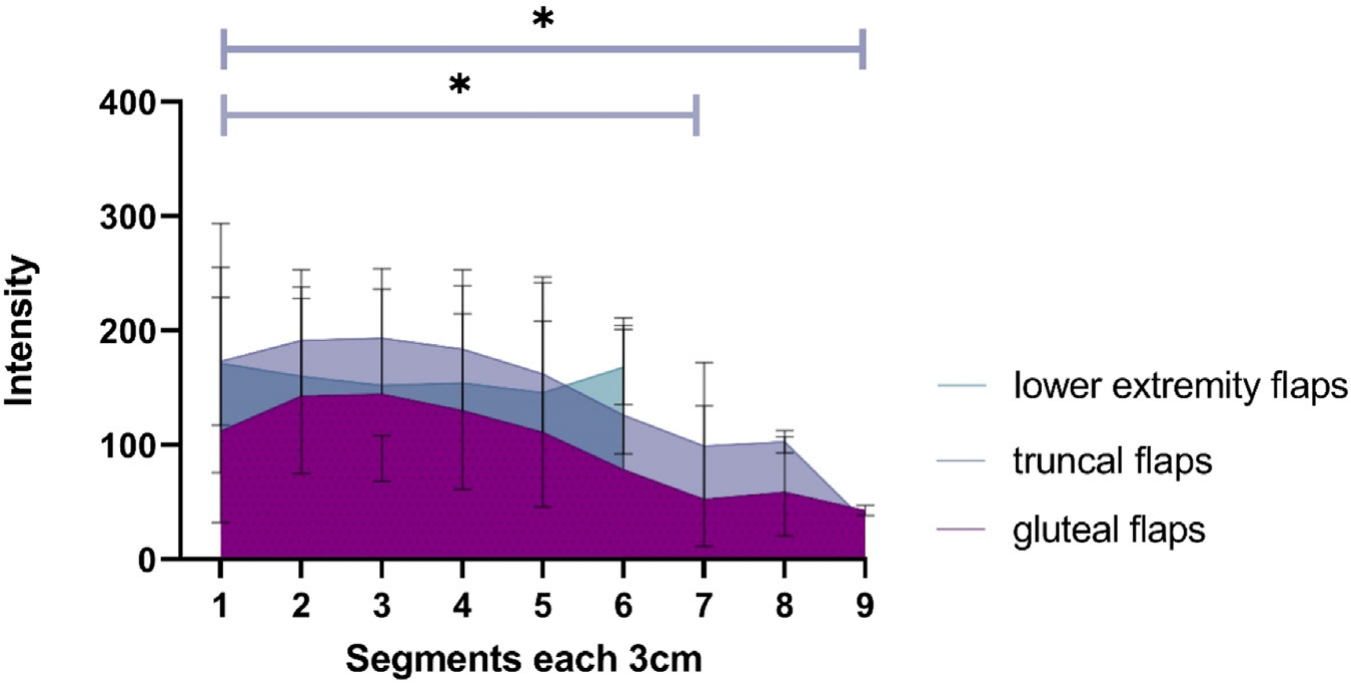
Perfusion analysis depending on flap region.

Subgroup analysis indicated anatomical location played a role in perfusion consistency. Lower extremity flaps had the most stable and uniform perfusion curves, with little deviation between contour thresholds. Gluteal and truncal flaps displayed greater perfusion heterogeneity, particularly beyond 18 cm distance from the pedicle (Fig. 4).

**Fig. 5:**
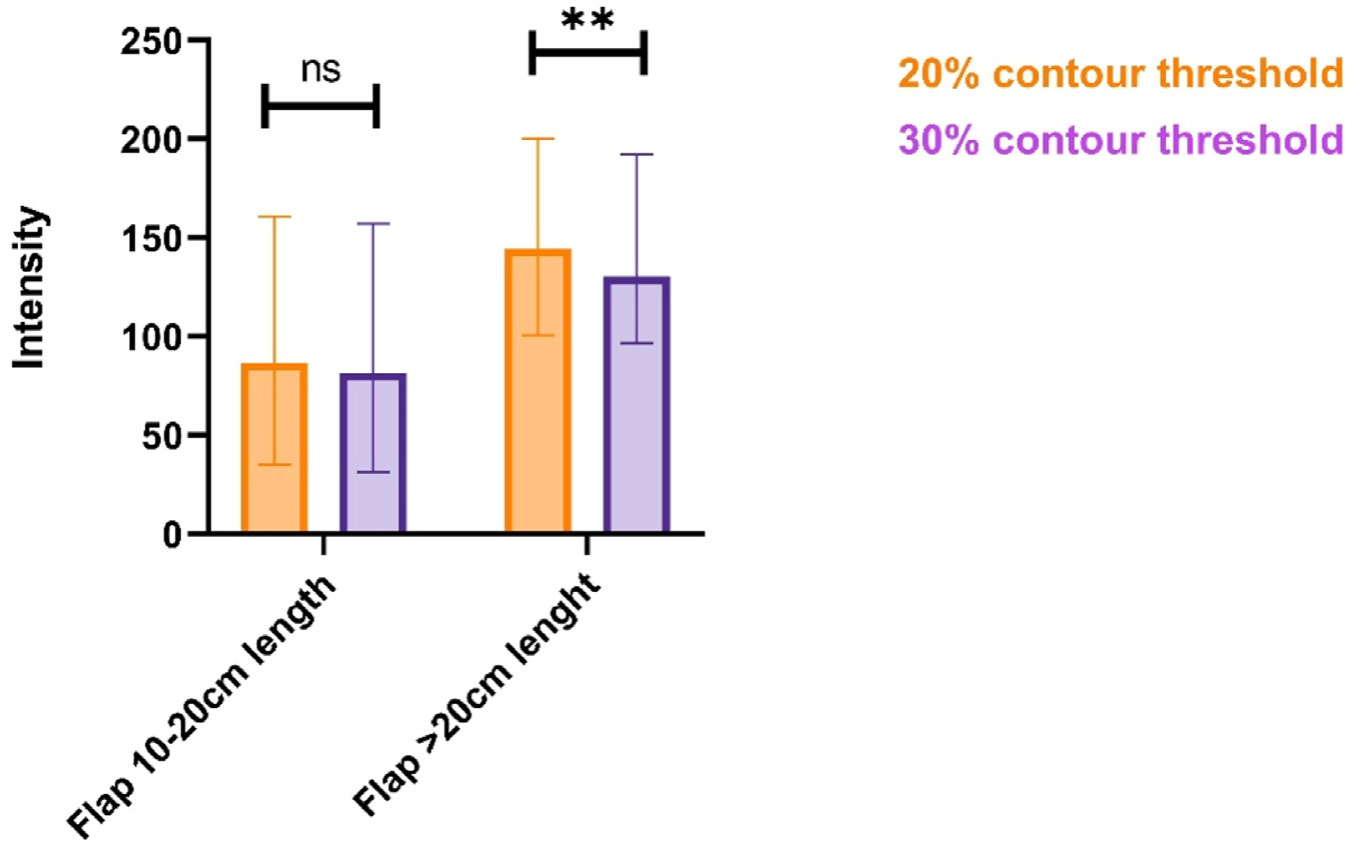
Flap perfusion analysis by flap length and fluorescence contour thresholds.

Evaluation of contour thresholds revealed that tissue areas within the 20% and 30% fluorescence contour levels consistently corresponded to clinically viable zones. No tissue loss occurred in regions within these thresholds, suggesting their reliability as intraoperative indicators for safe tissue preservation Fig. 5.

Additional exploratory analysis found that the decrease in perfusion from the proximal (segment 1) to the distal segments (segment 5–6) became statistically significant only in flaps >20 cm (figure 6), reinforcing the importance of intraoperative imaging for long flap designs. These findings highlight ICG angiography’s utility in identifying perfusion limits and optimizing flap safety margins during reconstructive planning.

The growing body of evidence around ∼30% normalized perfusion thresholds in related skin-flap contexts supports our contour findings, while emerging quantitative frameworks and reference perfusion curves promise improved inter-center comparability.

In summary, our results can be concluded as follows:

**Table:** 

**Flap Length**	Flaps <10 cm showed minimal perfusion drop (2.3 units), while >20 cm flaps showed significant decline, particularly after the 15 cm mark.
**Location**	Lower extremity flaps had the most uniform perfusion, followed by truncal and gluteal flaps.
**Contour Zones**	The 20% and 30% contour levels encompassed reliably perfused regions. All tissue within these thresholds survived without necrosis.

## Discussion

ICG fluorescence angiography provides a powerful adjunct for intraoperative decision-making in reconstructive flap surgery.^15^, ^16^, ^17^, ^18^, ^19^ By capturing real-time perfusion maps, it enhances the surgeon’s ability to identify at-risk tissue zones and adapt flap designs accordingly, especially diminishing the occurrence of fat necrosis.^16^^,^^20^, ^21^, ^22^

Advancements in intraoperative technologies that enable objective assessment of flap perfusion have the potential to significantly enhance outcomes in reconstructive microsurgery.^4^^,^^23^ Historically, surgeons have relied on clinical parameters—such as tissue colour, temperature, capillary refill, turgor, bleeding response, Doppler signal presence, and oxygen saturation—to evaluate perfusion. While these indicators remain valuable, they are not universally reliable. Their sensitivity may be limited, particularly in patients with very light or dark skin tones, or in flap types such as adipofascial or osseous flaps that lack a visible skin paddle, leading to ambiguous or misleading assessments.^19^^,^^24^ In cases where extended or large volume flaps are necessary, perfusion borders may be difficult to define increasing the risk for partial flap necrosis and revisional surgery. By using highly versatile perforator propeller flaps the axial torsion of the perforator pedicle up to 180° adds a possible risk factor for perfusion complications.

In this context, near-infrared fluorescence angiography using indocyanine green has emerged as a promising adjunct. ICG is a hydrophilic dye that rapidly binds to plasma proteins and fluoresces under near-infrared light, allowing dynamic, real-time visualization of perfusion and vascular flow.^25^ Owing to its intravascular confinement and rapid clearance, ICG provides a high-contrast, semi-quantitative view of tissue vascularity. An expanding body of literature supports its utility in plastic and reconstructive surgery, particularly in identifying perfusion deficits and guiding intraoperative decisions.^21^

At our institution, we have adopted ICG fluorescence angiography from 2013 on and utilized this technique first in free flap surgery, especially in DIEP flap breast reconstruction.^16^^,^^26^ We then extended the use as well to other free flaps, including muscle flaps, initially specifically in such scenarios where conventional clinical evaluation was inconclusive.^17^^,^^19^^,^^27^ Based on our experience with several hundreds of flaps we could prove that ICG angiography enhances decision-making in free and pedicled flap surgery, offering a versatile and reproducible approach to optimize flap outcomes. In line with the findings of Moyer et al.^28^ and Ludolph et al.,^16^ this study suggests that the 20% and 30% contour levels serve as reliable indicators of adequate tissue perfusion in PPFs. Importantly, all flaps in this series that met these criteria survived without tissue loss. This threshold also aligns with recent prospective data in breast and extremity reconstruction, where relative perfusion values around 30% contour level have shown predictive validity for viability.^29^ The absence of partial necrosis despite three cases of postoperative venous congestion is noteworthy. This finding may reflect the fact that venous outflow compromise does not inevitably result in tissue loss when recognized and treated promptly. There is evidence, that insufficient flap enhancement on final intraoperative ICG angiography can precede the clinical manifestation of venous congestion and may predict subsequent flap compromise.^30^ However venous congestion was intraoperativly not foreseable in our cases, thereby enforcing that intraoperative ICG angiography primarily depicts perfusion at the time of image acquisition and may not fully capture delayed postoperative alterations in venous drainage occurring after flap inset and tissue edema.

Longer perforator propeller flaps showed expected perfusion drops, particularly beyond 15 cm flap length. This observation supports the concept of choke vessel zones and inter-perforator variability, as described by Taylor and Saint-Cyr. Importantly, all flaps in the present cohort survived without partial necrosis, despite the inclusion of extended flap designs up to 27 cm. This observation highlights the potential of ICG angiography to support individualized flap planning by objectively visualizing perfusion boundaries rather than relying solely on empirical length guidelines.

Notably, lower extremity flaps showed the most consistent perfusion patterns, possibly due to dense perforator networks. Truncal and gluteal flaps, especially those with higher adipose content, exhibited more variation and underscore the value of ICG imaging.

While our analysis was based on contour levels, suggesting that a 20–30% threshold performed well, several recent studies advocate for complementary dynamic perfusion parameters such as ingress slope, time-to-peak, and washout time that correlate with tissue oxygenation and clinical outcomes. These quantitative metrics may enhance precision in borderline zones and aid in detecting venous congestion or inadequate outflow beyond static intensity cut-offs.^29^ Because perforator propeller flaps have only scarcely been investigated so far with ICG imaging.^13^ there are some limitations, which include the retrospective design or absence of volumetric analysis. A further limitation is the absence of a control group undergoing conventional clinical perfusion assessment without ICG angiography. Howeber since ICG angiography is part of the established intraoperative decision-making process at our institution, a contemporaneous control group without ICG assessment is not available, and deliberately withholding perfusion imaging would have raised ethical concerns regarding patient safety. Nevertheless, recent studies in implant-based breast reconstruction have identified normalized perfusion thresholds of approximately 30% as clinically relevant predictors of mastectomy skin flap viability. These findings are consistent with our observation that tissue areas within the 20–30% fluorescence contour levels remained clinically viable, suggesting that similar perfusion thresholds may have relevance across different reconstructive settings while also underlining the search and need for flap-specific calibration.^31^^,^^32^ Future directions could include automated image analysis, integration of 3D perfusion models, and prospective trials to define standardized perfusion cut-offs. In addition it is known that ICG signals can be influenced by vasopressors, temperature, tissue thickness/adiposity, edema, and ambient light, so standardized anaesthetic and optical conditions remain advisable. Thick adipose/dermis may attenuate emitted fluorescence, arguing for internal normalization (reference ROI) when comparing segments.^33^

A key barrier to wider adoption hitherto remains the lack of standardized acquisition and interpretation protocols.^34^ Expert consensus and dosing guidance (e.g., ISFGS) emphasize consistent ICG dosing, inflow/egress timing, camera-to-tissue working distance, and ROI normalization to reduce inter-case/device. Nevertheless growing current clinical practice data aligning with our findings show that ICG angiography provides real-time, actionable data that enhances the safety and planning of PPFs, especially in anatomically variable or extended flap designs.

## Conclusion

ICG fluorescence angiography is a valuable tool in the intraoperative assessment of PPFs. Perforator propellor flaps with a total length <10 cm and those confined within the 30% perfusion contour are associated with optimal outcomes. These findings strongly support integrating ICG imaging into surgical planning for enhanced flap safety and precision.

Moreover, incorporating standardized acquisition protocols and combining static contour mapping with dynamic perfusion metrics may further improve the predictive accuracy of ICG angiography in flap surgery. The expanding role of automated image analysis and perfusion modelling offers additional promising directions for future research and clinical application. While the retrospective design and limited sample size represent inherent limitations, the present data contribute to the growing body of evidence supporting the clinical utility of ICG imaging in reconstructive surgery. Future prospective multicenter studies integrating dynamic perfusion parameters and automated image analysis may further refine perfusion thresholds and facilitate the development of standardized intraoperative decision-making algorithms.

## Ethics approval

This study was approved by the Ethics Committee of the Friedrich-Alexander University, Erlangen (Approval No 424–18B) and the study was conducted in accordance with the Declaration of Helsinki.

## Funding

None.

## Declaration of competing interest

The authors declare that they have no conflict of interest.

## Appendix Supplementary materials

Image, application 1
