# Supplementary material for: Exploring perfusion limits in pedicled perforator propeller flaps via intraoperative indocyanine green angiography
Source: JPRAS Open. 2026 Jul 22;51:584–93. doi: 10.1016/j.jpra.2026.07.016 (PMC13485620; doi:10.1016/j.jpra.2026.07.016)
Supplement: Supplementary file 1 [file mmc1.docx]

*Supp.1:* Overview of the patient cohort

| Case-ID | Age (years) | Defect | | | Postoperative complications | |
| --- | --- | --- | --- | --- | --- | --- |
|  |  | **Cause** | **Location** | **Defect coverage** | **Overview** | **Flap partial necrosis / loss** |
| 1 | 49 | Status post bilateral mastectomy | paravertebral | D-ICAP | - | - |
| 2 | 43 | Crohn’s disease, fistula | sacrovaginal | S-GAP | Venous congestion, revision | - |
| 3 | 34 | Chronic ulcer | sacral | S-GAP | Venous congestion | - |
| 4 | 72 | Pressure sore | gluteal + prasacral | S-GAP | - | - |
| 5 | 50 | Status post dermatofibrosarcoma resection | paravertebral | D-ICAP | - | - |
| 6 | 76 | Status post leiomyosarcoma resection | sacral | S-GAP | - | - |
| 7 | 84 | Status post melanoma resection | paravertebral | D-ICAP | - | - |
| 8 | 42 | Ulcerative colitis, chronic fistula after rectal extirpation | sacral | S-GAP | Wound infection | - |
| 9 | 41 | Status post dermatofibrosarcoma resection | left thoracic region | L-ICAP | - | - |
| 10 | 81 | Status post melanoma resection | paravertebral | D-ICAP | - | - |
| 11 | 73 | Pressure sore | sacral | S-GAP | - | - |
| 12 | 62 | Status post ankle fracture after fall | right lateral malleolus | A. fibularis | - | - |
| 13 | 55 | Status post melanoma resection | right thoracic region | L-ICAP | - | - |
| 14 | 63 | Status post melanoma resection | left thigh | A. prof. femoris | - | - |
| 15 | 75 | Status post rectal extirpation for rectal carcinoma | sacral | S-GAP | - | - |
| 16 | 56 | Status post rectal extirpation for rectal carcinoma, perianal fistula | sacral | I-GAP | - | - |
| 17 | 64 | Status post BCC resection | paravertebral | D-ICAP | - | - |
| 18 | 36 | Crohn’s disease, fistula | rectovaginal | I-GAP | Wound infection | - |
| 19 | 69 | Pressure sore | sacral | S-GAP | - | - |
| 20 | 24 | Status post melanoma resection | left thoracic region | L-ICAP | - | - |
| 21 | 69 | Status post melanoma resection | medial plantar foot left | A. plantaris medialis | - | - |
| 22 | 87 | Pressure sore | paravertebral | D-ICAP | - | - |
| 23 | 58 | Status post melanoma resection | paravertebral | D-ICAP | - | - |
| 24 | 79 | Status post squamous cell carcinoma resection | gluteal | S-GAP | - | - |
| 25 | 78 | Status post BCC resection | pretibial right | A. tibialis posterior | Wound infection | - |
| 26 | 81 | Pressure sore | gluteal | I-GAP | Acute hematoma formation, revision | - |
| 27 | 60 | Status post desmoid tumor resection | paravertebral | D-ICAP | - | - |
| 28 | 36 | Status post Achilles tendon rupture | pretibial right | A. tibialis posterior | - | - |
| 29 | 44 | Status post resection of infected atheromas | paravertebral | D-ICAP | - | - |
| 30 | 25 | Status post pilonidal sinus | praesacral | S-GAP | - | - |
